# Intestinal Piezo1 aggravates intestinal barrier dysfunction during sepsis by mediating Ca^2+^ influx

**DOI:** 10.1186/s12967-024-05076-z

**Published:** 2024-04-04

**Authors:** Zimeng Yan, Lei Niu, Shangyuan Wang, Chengjin Gao, Shuming Pan

**Affiliations:** 1 Department of Emergency, Xinhua Hospital Affiliated to Shanghai Jiao Tong University School of Medicine, Yangpu District, Shanghai, China; 2 Department of Emergency, Shanghai Jiahui International Hospital, No. 689, Guiping Rd., Shanghai, China

**Keywords:** Sepsis, Piezo1, Intestinal barrier, Ca2+ influx

## Abstract

**Introduction:**

Intestinal barrier dysfunction is a pivotal factor in sepsis progression. The mechanosensitive ion channel Piezo1 is associated with barrier function; however, its role in sepsis-induced intestinal barrier dysfunction remains poorly understood.

**Methods:**

The application of cecal ligation and puncture (CLP) modeling was performed on both mice of the wild-type (WT) variety and those with Villin-Piezo1^flox/flox^ genetic makeup to assess the barrier function using in vivo FITC-dextran permeability measurements and immunofluorescence microscopy analysis of tight junctions (TJs) and apoptosis levels. In vitro, Caco-2 monolayers were subjected to TNF-α incubation. Moreover, to modulate Piezo1 activation, GsMTx4 was applied to inhibit Piezo1 activation. The barrier function, intracellular calcium levels, and mitochondrial function were monitored using calcium imaging and immunofluorescence techniques.

**Results:**

In the intestinal tissues of CLP-induced septic mice, Piezo1 protein levels were notably elevated compared with those in normal mice. Piezo1 has been implicated in the sepsis-mediated disruption of TJs, apoptosis of intestinal epithelial cells, elevated intestinal mucosal permeability, and systemic inflammation in WT mice, whereas these effects were absent in Villin-Piezo1^flox/flox^ CLP mice. In Caco-2 cells, TNF-α prompted calcium influx, an effect reversed by GsMTx4 treatment. Elevated calcium concentrations are correlated with increased accumulation of reactive oxygen species, diminished mitochondrial membrane potential, and TJ disruption.

**Conclusions:**

Thus, Piezo1 is a potential contributor to sepsis-induced intestinal barrier dysfunction, influencing apoptosis and TJ modification through calcium influx-mediated mitochondrial dysfunction.

**Supplementary Information:**

The online version contains supplementary material available at 10.1186/s12967-024-05076-z.

## Introduction

Sepsis is a severe systemic inflammatory reaction in the body, and an impaired response to infection leads to organ dysfunction [1, 2]. Approximately 31.5 million new cases and 5.3 million fatalities are reported each year globally [1, 3]. The main pathophysiology of sepsis involves systemic bacterial translocation associated with multiple organ dysfunction syndrome (MODS) due to intestinal barrier dysfunction [4, 5]. Gut monolayer epithelial cells serve as selective barriers, preventing the transfer of potentially harmful pathogens, toxins, and antigens from the lumen to the mesenteric lymph nodes and circulation [6]. Therefore, it is crucial to address the intestinal barrier dysfunction in patients with sepsis.

Maintenance of intestinal barrier function relies heavily on the tight junctions (TJs) present in intestinal epithelial cells. During sepsis, exposure to locally migrating bacteria and endotoxins triggers the local activation of intestinal mucosal immunity, leading to increased intestinal permeability through alterations in TJs [7, 8]. Experimental sepsis studies have shown redistribution of compact proteins, such as occludin and claudin-1, 3, 4, 5, and 8 [9]. In addition, epithelial apoptosis caused by inflammation plays a significant role in sepsis progression. Mouse sepsis models and autopsy studies of patients with sepsis have demonstrated a significant increase in intestinal epithelial/lymphocyte apoptosis compared to non-septic deaths, which are associated with barrier function disruption [10].

Cells of the intestinal mucosa display remarkable mechanical sensitivity, sensing mechanical cues from the external milieu and translating them into biochemical signals that govern their functionality [11]. Piezo1, a mechanosensitive protein discovered in 2010, responds to mechanical stimulation by promoting the influx of calcium into the cytoplasm [12]. Piezo1 is widely expressed in various tissues and regulates multiple physiological and pathological processes, including proliferation, differentiation, adhesion, migration, and apoptosis [13]. However, its role in intestinal barrier dysfunction during sepsis remains unclear.

The central conjecture of this investigation is that Piezo1 contributes to the elevation of intracellular calcium levels and the induction of mitochondrial dysfunction, ultimately promoting intestinal barrier dysfunction in sepsis by disrupting TJs and increasing apoptosis. In the guts of cecal ligation and puncture (CLP) mice, we observed an association between Piezo1 expression and decreased TJ expression, as well as increased apoptosis in epithelial cells. In Caco-2 cells, Piezo1 induces calcium influx, leading to mitochondrial dysfunction, which may also be associated with decreased TJ expression.

## Method

### Chemicals and antibodies

4 kDa FITC-dextran was obtained from Maokang Biotechnology (Shanghai, China). Roswell Park Memorial Institute (RPMI) 1640 medium and Dulbecco’s Modified Eagle Medium (DMEM) was procured from Gibco (USA). Antibodies for Piezo1, Occludin and ZO-1 were procured from Proteintech (China). The following chemicals were obtained from Servicebio (China): fluorescein (FITC) Tunel Cell Apoptosis Detection Kit, Phosphate Buffered Saline (PBS) and Hank’s Balanced Salt Solution (HBSS). Fluorescent-conjugated antibodies for flow cytometry were procured from BD Bioscience (USA). Secondary antibodies conjugated with AlexaFluor-488 or Alexa Fluor 594, Annexin V-FITC /PI Apoptosis Detection Kit, JC-1 Mitochondrial Membrane Potential Assay Kit, SYBR qPCR Master Mix, Rhod-2 AM and Fetal Bovine Serum (FBS) were procured from Yeason (China). Yoda1 and MitoSOx Red were procured from MedChemExpress (USA)**.** MitoTEMPO was acquired from Topscience (China). DMEM no Ca^2+^ was obtained from Meiluncell (China).

### Mice

Six to eight-week-old WT C57BL/6 mice were procured from Shanghai Slake Laboratory Animal Company. Villin-CreERT and Piezo1^flox/flox^ mice were provided by Shanghai Nanfang Mode Biotechnology Co., Ltd, and their identification was conducted via PCR using tail samples. All animals were maintained in specific pathogen-free conditions, with a 12-h light/12-h dark cycle at 25 °C, and provided with ad libitum access to food and water. Experiments commenced after a one-week acclimatization period for the mice. The experimental procedures adhered to in this study were sanctioned by the Animal Studies Committee of Xinhua Hospital Affiliated To Shanghai Jiao Tong University School Of Medicine. All procedures performed in studies involving animals were in accordance with the ethical standards of the institution or practice at which the studies were conducted.

### Mice CLP model establishment

The CLP method, previously validated in a separate study [14], was employed to establish the sepsis mice model. Mice were anesthetized using intraperitoneal injection of tribromoethanol (10 mg/kg). The cecum underwent ligation and puncture procedures utilizing a 21-gauge needle. This was succeeded by the evacuation of cecal contents, repositioning of the cecum, and closure of the incision through muscle and skin layers. In the subsequent steps, the mice received a subcutaneous injection of 1 ml of saline at 37 °C, after which they were rehoused in their respective cages.

### Intestinal barrier permeability analysis

After 24 h of CLP modeling, the mice underwent a three-hour fast. To ensure sterility, a gavage needle was autoclaved, and then 150 µl of 80 mg per milliliter four-kilodalton FITC dextran in sterile water was administered via gavage. Serum samples (0.1 ml) were collected four hours after oral gavage, diluted five-fold, and measured using an H4 microplate reader with fluorescence read at an excitation of 485 nm and an emission of 528 nm.

### Real-time quantitative polymerase chain reaction

TRIzol reagent (Takara, Japan) was utilized for the extraction of total RNA from both intestinal tissues and cells.RNA integrity was verified using a Nanodrop Spectrophotometer, followed by reverse transcription to complementary DNA using PrimeScript™ RT reagent Kit (Takara, Japan) for qPCR. Real-time quantitative polymerase chain reaction (qPCR) was performed using the SYBR qPCR Master Mix on a Q3 instrument. Normalization of gene expression data was carried out using GAPDH, and the outcomes were represented as 2^−ΔΔCt^. The specific primer oligonucleotide sequences utilized in this investigation are detailed in Table 1.

**Table 1:** The primer sequence for RT-qPCR

Genes	Sequence of primers (5ʹ-3ʹ)
GAPDH (mouse)	F: AGGTCGGTGTGAACGGATTTG; R: TGTAGACCATGTAGTTGAGGTCA
Piezo1 (mouse)	F: AGGACTTCCCCACCTATTGG; R: CCAGGGATGAGGATACTGGAAAA

### Intestinal tissue sample preparation

Mice were euthanized using pentobarbital sodium anesthesia. The intestine adjacent to the cecum was extracted and divided into upper and lower sections. The bottom section was subjected to fixation in a solution of 4% paraformaldehyde to facilitate subsequent sectioning and staining procedures. In contrast, the upper section was preserved by storing it at a temperature of − 80 °C.

### Hematoxylin–eosin staining

The tissue was fixed using paraformaldehyde, subsequently embedded in paraffin, and sectioned into 8-μm-thick slices for HE staining.

### 16S rRNA sequencing

Mouse fecal and lung tissue samples were systematically collected for this study. Primers were meticulously designed, targeting conserved regions, to amplify single or multiple variable regions of the rRNA gene within each sample. The ensuing PCR product underwent a purification process, succeeded by a second round of PCR amplification, pooling, library purification, on-machine sequencing, and Miseq PE300 sequencing. To process the obtained high-quality sequences, Usearch software was employed for de-chimerization, merging, and division into operational taxonomic units (OTUs) based on a 97% sequence similarity threshold. Subsequently, the most abundant sequence in each OTU was selected as its representative sequence for further analysis. α Diversity was comprehensively evaluated. The QIIME software facilitated the derivation of composition and abundance distribution tables for each sample, and the results were elegantly presented through histograms.

### TUNEL staining

Sections derived from tissues that had been fixed in paraformaldehyde and subsequently embedded in paraffin were subjected to a deparaffinization process utilizing xylene, followed by a rehydration procedure through a series of ethanol gradients. In order to detect apoptosis, a TUNEL Apoptosis Detection Kit was utilized in accordance with the instructions provided by the manufacturer.

### Enzyme-linked immunosorbent assay

Mouse IL (interleukin)-18 and IL-10 levels were measured and quantified employing an enzyme-linked immunosorbent assay (ELISA) kit (Aifang, China) following the manufacturer’s protocol.

### Isolation of colonic lamina propria cells and flow cytometry

The intestines were isolated and longitudinally opened, with subsequent removal of feces, fat tissues, and Peyer's patches using forceps. These intestines were then divided into 2-cm-long segments and cleansed with PBS. The segments were immersed in a solution of FBS (50x) supplemented with dithiothreitol (1 mM) and EDTA (30 mM), and subjected to incubation at 37 °C and 250 r.p.m. for 20 min, followed by a repeated iteration of this process. After filtration of the suspensions, the remaining tissues were further cut into 0.5-cm-long fragments and digested in an incubator at 37 °C and 250 r.p.m. for 50 min using RPMI 1640 medium supplemented with 10% FBS and collagenase IV. The digested solution underwent filtration, and the resulting mixture was centrifuged at 500×*g* for 5 min at 4 °C to concentrate live mononuclear cells. The fluorescent-conjugated antibodies were used to label lamina propria cells, which were then subjected to analysis using flow cytometry. For intracellular staining, cells were both fixed and permeabilized with a fixable buffer, and data collection was performed through flow cytometry. M1 macrophages were defned as CD45^+^CD11b^+^F4/80^+^CD86^+^ cells, M2 macrophages were defned as CD45^+^CD11b^+^F4/80^+^CD206^+^ cells.

### Cell culture

The human colonic adenocarcinoma cell line Caco-2 was procured from Kuisai Biotechnology (Shanghai, China). Cells were seeded in T25 flasks (NEST, Suzhou, China) with DMEM medium containing 10% FBS and 1% penicillin/streptomycin (Sigma-Aldrich, MO, USA). Cell culture was maintained in an incubator at 37 °C and 5% CO_2_. To simulate damage to the intestinal mucosal barrier, epithelial cell monolayers underwent TNF-α (10 ng/ml) (PeproTech, USA). Furthermore, to modulate Piezo1 activation, Caco-2 cells were treated with 5 μM GsMTx4 (Selleck, China) to inhibit Piezo1.

### Immunochemistry

Caco-2 cells were cultivated on glass slides for a period of 14 days. Following this, the slides underwent a 5-min PBS wash and were then immobilized with 4% paraformaldehyde dissolved in PBS. In the case of colon tissue examination, 8-μm sections were subjected to two 10-min dewaxing treatments with xylene, followed by a stepwise rehydration process involving various ethanol concentrations (100%, 95%, 80%, and 75%, 5 min each). Antigen retrieval was executed by exposing the slices to a 0.01 M sodium citrate buffer at 100 °C for 2 min. Subsequently, the slices were blocked with 5% Goat Serum for 1 h, followed by overnight incubation with primary antibodies at 4 °C. Following three PBS washes, secondary antibodies were administered at 37 °C for 1 h. Nuclei were counterstained using DAPI, and images were captured with an Olympus camera. Quantitative analysis was conducted using ImageJ software version 1.8.0.112 (Bethesda, USA). The antibodies used were anti-Piezo1 (1:1000), anti-ZO-1 (1:1000), and anti-Occludin (1:1000).

### Calcium imaging

Prior to TNF-α incubation and loading with 5 μM Fluo-4A (Beyotime, China), Caco-2 cells were pretreated with 2.5 μM GsMTx4. This step was conducted under dark conditions at 37 °C for a duration of 30 min. Afterward, the cells were rinsed three times with Hank’s Balanced Salt Solution, and observations were conducted utilizing an Olympus camera. Following this, the introduction of 20 μM Yoda1 occurred once the baseline fluorescence value had reached a stable state. Mitochondrial Ca^2+^ levels were assessed utilizing Rhod-2 AM. Cells underwent incubation with 5 μM Rhod-2 AM and HBSS buffer following the reagent manufacturer’s guidelines. The staining process was conducted at 37 °C for 30 min in a light-protected environment, after which the samples were imaged using a fluorescence microscope.

### Cell apoptosis analysis

Annexin V-FITC/PI Apoptosis Detection Kit was employed for cell apoptosis measurement. Cells were seeded at a density of 1 × 10^6^ cells per well in 6 cm plates and cultured until the formation of the intestinal epithelial barrier, followed by 24-h TNF-α incubation. The procedures were carried out following the manufacturer’s guidelines. Each cell sample was treated with 5 μl Annexin V-FITC and 10 μl PI, incubated in the dark at 25 °C for 15 min, and then analyzed through flow cytometry (Beckman Coulter, USA). All experiments were performed in triplicate. Cells positive for both Annexin V-FITC and PI were considered as undergoing late apoptosis, while those negative for both indicated live cells. Cells positive for PI and negative for Annexin V-FITC were classified as necrotic cells, and those negative for PI but positive for Annexin V-FITC were considered early apoptotic cells.

### Mitochondrial O_2_ measurement

To assess mitochondrial O2, MitoSOX Red was employed. After two washes with PBS, cells were subjected to an incubation at a temperature of 37 °C for a duration of 30 min with the presence of 5 μM MitoSOX Red and subsequently imaged using an Olympus camera.

### Mitochondrial membrane potential measurement

The mitochondrial membrane potential was assessed by using a JC-1 mitochondrial membrane potential assay kit. Following two PBS washes, cells were labeled at 37 °C for 20 min with JC-1 working solution. Imaging for JC-1 monomers and polymers was done using an Olympus camera.

### Statistical analysis

All statistical analyses were conducted using GraphPad Prism 9.0 software. The results are expressed as the mean ± standard error of the mean (SEM). Comparisons between two groups were carried out using an unpaired, two-tailed Student’s t-test. A significance level of P < 0.05 was considered statistically significant for all data.

## Result

### Piezo1 participates in the impairment of the intestinal barrier of CLP-induced sepsis mice

**Fig. 1:**
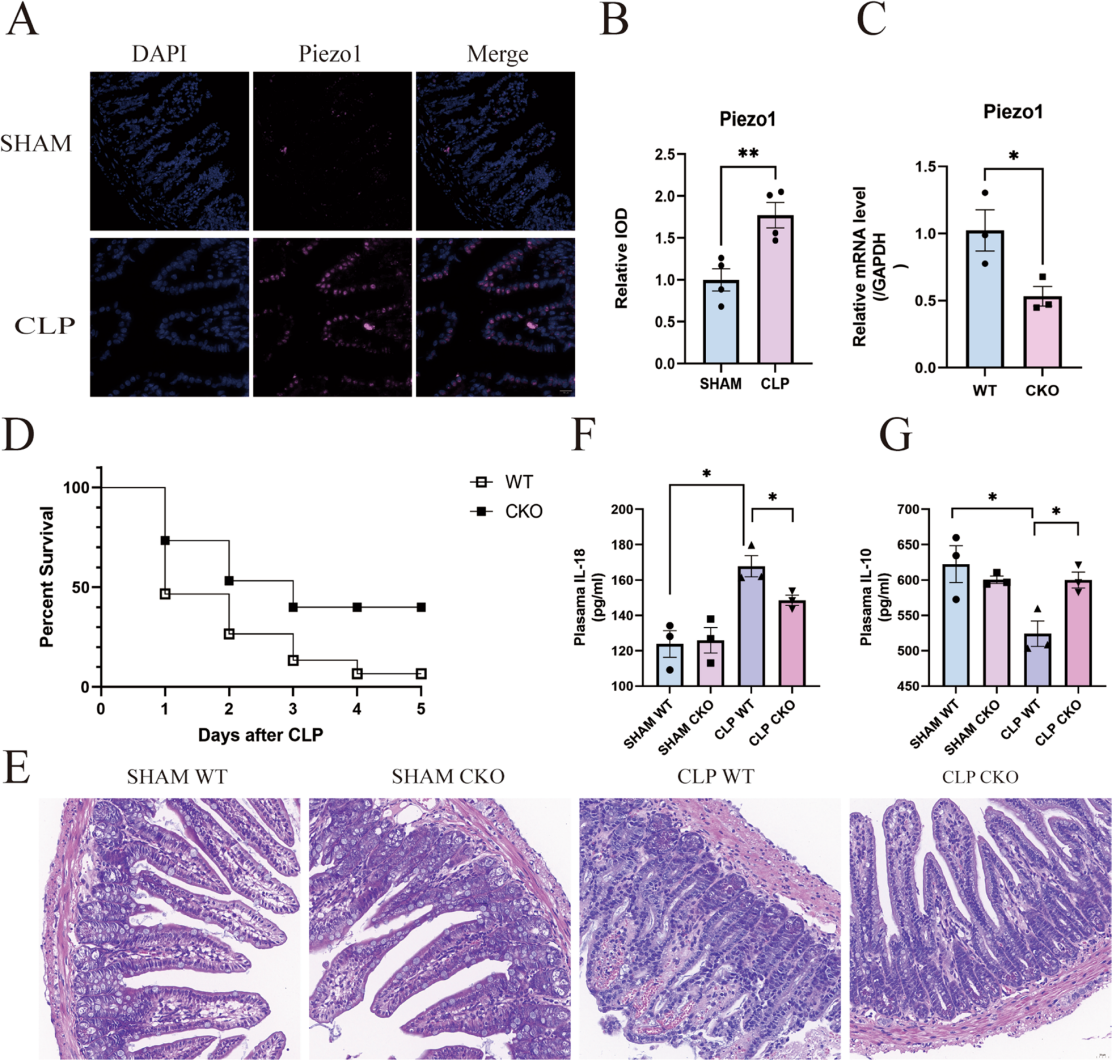
Piezo1 participates in the impairment of the intestinal barrier of CLP-induced sepsis mice. **A** Immunofluorescence analysis of Piezo1 in the intestinal tissues of mice with normal conditions and mice with sepsis. Scale bar 50 μm. **B** Quantitative analysis of immunofluorescence evaluation was conducted (n = 4 for each group). **C** RT-PCR was employed to analyze Piezo1 expression in the ileum of both Villin-Piezo1flox/flox mice and WT mice (n = 3 for each group). **D** Survival for 7 days in WT and CKO mice afer CLP treatment (n = 15 for each group). **E** Evaluation of intestinal pathological alterations was accomplished through H&E staining. Scale bar 50 μm (n = 4 for each group). Enzyme-linked immunosorbent assay (ELISA) was employed to ascertain the levels of IL-18 (**F**) and IL-10 (**G**) in plasma (n = 3 for each group)

Piezo1 expression was examined using immunofluorescence staining in intestinal tissues obtained from mice under normal conditions and from mice with CLP-induced sepsis. Compared with normal mice, Piezo1 protein levels were significantly elevated in the intestinal tissues of mice with CLP-induced sepsis (Fig. 1A, B). To investigate the role of Piezo1 in intestinal barrier injury in mice with CLP-induced sepsis, we generated tamoxifen-inducible homozygous Piezo1 conditional knockout mice with gut-specific villin-CreERT and Piezo1^flox/flox^ allele expression. Confirmation of gut-specific Piezo1 deletion was achieved through RT-PCR analysis (Fig. 1C), demonstrating the successful knockout of Piezo1 in the gut. After CLP, the 7-d mortality rate of wild-type (WT) mice was 93.33%, whereas that of Villin-Piezo1^flox/flox^ (CKO) mice was 60% (Fig. 1D). Hematoxylin-eosin staining (HE) revealed that under basal conditions, neither WT nor CKO mice displayed evident pathological manifestations in the intestine. However, following CLP, CKO mice exhibited significantly less epithelial damage, neutrophil infiltration, and crypt architecture disruption than their WT littermates (Fig. 1E). Moreover, CLP markedly elevated the levels of the plasma pro-inflammatory cytokine IL-18 and reduced the expression of the mucosal anti-inflammatory cytokine IL-10 in WT mice following CLP treatment. Notably, Piezo1 knockout exerted a notable mitigating effect on the regulation of these cytokines (Fig. 1F, G). We performed 16sRNA sequencing of the lung tissue and feces of mice and found that after CLP, the α diversity in the lung tissue of WT mice increased and the α diversity in the lung tissue of CKO mice also increased, but to a lesser extent than that of WT mice, however, there was no statistical difference. In addition, we found that bacteria that were not present in the lung tissue of the SHAM group were found in the lung tissue of WT mice after CLP, and these bacteria were also detected in the feces of the same group, but they were not found in CKO mice, such as Verrucomicrobiota. These indicated that bacterial translocation may have occurred after CLP, but Piezo1 knockdown mitigated bacterial translocation. These findings collectively imply the potential involvement of Piezo1 in the intestinal barrier impairment during CLP-induced sepsis (Additional file 1: Figure S1).

### Villin-Piezo1^flox/flox^ mice demonstrate resilience against CLP-induced disruption of the intestinal barrier

**Fig. 2:**
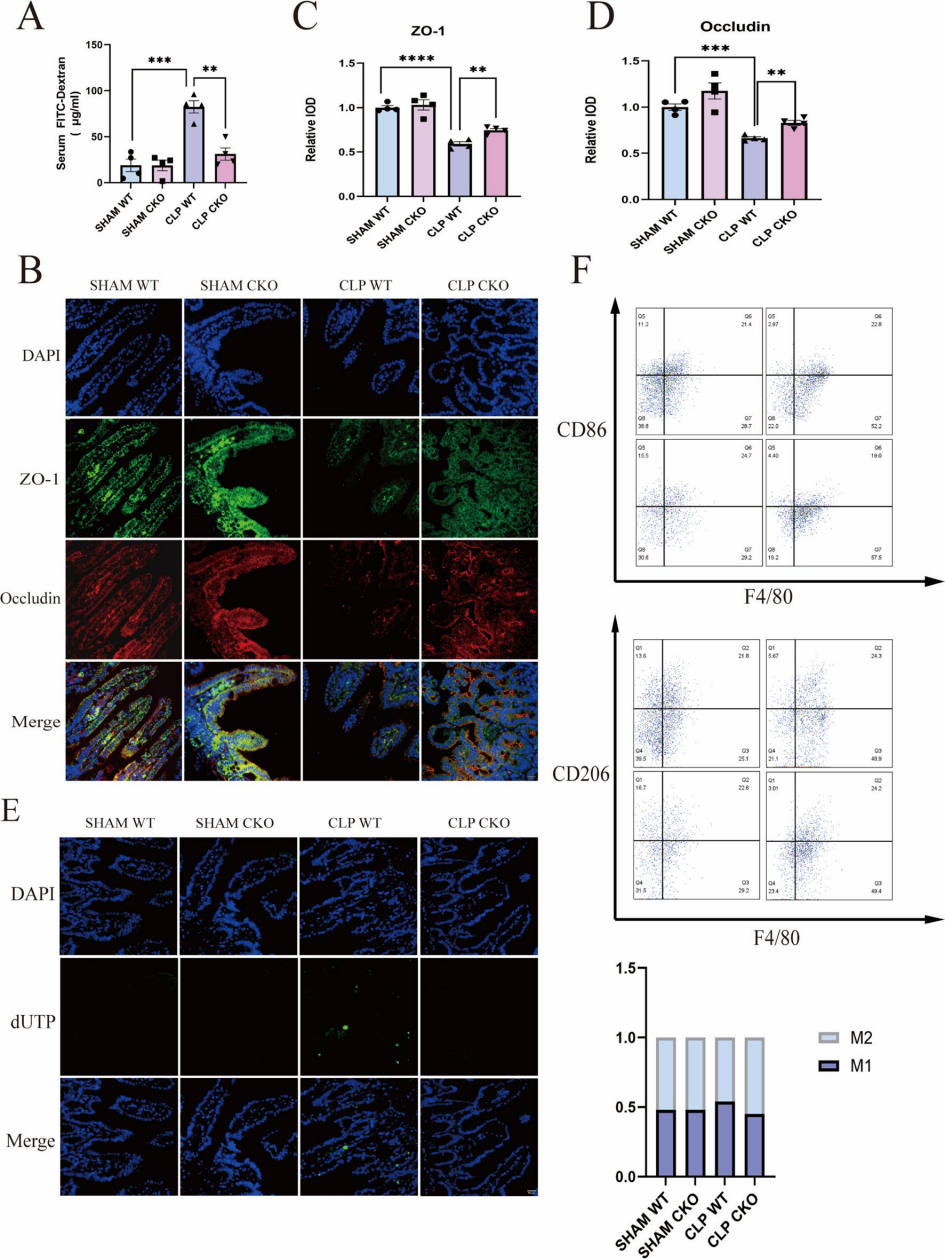
Villin-Piezo1^flox/flox^ mice demonstrate resilience against CLP-induced disruption of the intestinal barrier. **A** Intestinal permeability is detected by administering FITC-dextran in plasma (n = 4 for each group). **B**–**D** Immunofluorescence analysis of ZO1 and Occludin in the intestinal tissues of mice, and quantitative analysis of immunofluorescence evaluation (n = 4 for each group). Scale bar, 50 μm. **E** Section of intestines were subjected to TUNEL staining. Scale bar, 50 μm (n = 4 for each group). **F** M1 and M2 macrophages were identified with flow cytometry with markers of CD86F4/80 and CD206F4/80, respectively (n = 3 for each group)

In sepsis, a decline in the expression of TJs within the intestine ensues, precipitating the breakdown of the intestinal barrier, which, in turn, exacerbates the systemic inflammatory response. To gain deeper insights into the role of Piezo1 in this scenario, we assessed intestinal permeability in mice through intragastric administration of FITC-dextran 24 h after CLP. Remarkably, CLP-treated mice exhibited increased serum FITC-dextran levels, a marker of heightened intestinal permeability, compared with the control group. However, a substantial reduction in FITC-dextran levels was observed in Villin-Piezo1^flox/flox^ mice, implying that the deletion of Piezo1 ameliorated the exacerbated intestinal permeability triggered by CLP (Fig. 2A). Next, we investigated the influence of Piezo1 on the disruption of TJs induced by CLP. Evidently, the expression of essential TJ proteins, ZO-1 and occludin, underwent a notable decreased in WT mice subjected to CLP compared to that in the control group. Strikingly, Piezo1 deficiency effectively curbed the reduction in TJ protein expression induced by CLP, thereby preserving their levels (Fig. 2B–D). These outcomes robustly indicate the potential involvement of Piezo1 in facilitating intestinal mucosal barrier compromise in sepsis, potentially through its regulatory influence on TJ protein degradation. Importantly, the disruption of the intestinal mucosal barrier is intricately linked with the phenomenon of epithelial cell apoptosis. In this context, significant accumulation of TUNEL-positive cells was discernible in the colons of WT mice subjected to CLP. Conversely, the colon tissue of Villin-Piezo1^flox/flox^ mice exhibited a marked reduction in TUNEL-positive signals, indicating the potential attenuation of epithelial cell apoptosis (Fig. 2E).

CLP triggers the polarization of intestinal macrophages toward the M1 phenotype, thereby contributing to the disruption of the intestinal barrier [15]. Conversely, upon the amelioration of intestinal damage induced by lipopolysaccharide (LPS), the proportion of M2 macrophages increases. To delve deeper into the influence of Piezo1 on macrophage polarization, we assessed its effects. CLP treatment in WT mice resulted in an augmentation of M1 macrophages within the gut, accompanied by a reduction in the proportion of M2 phenotype macrophages. Furthermore, the deficiency of Piezo1 significantly reduced the proportion of M1 macrophages while up-regulating the M2 population (Fig. 2F).

### Piezo1 plays a pivotal role in the disruption of the intestinal barrier induced by TNF-α leading to increased degradation of TJ proteins and apoptosis

**Fig. 3:**
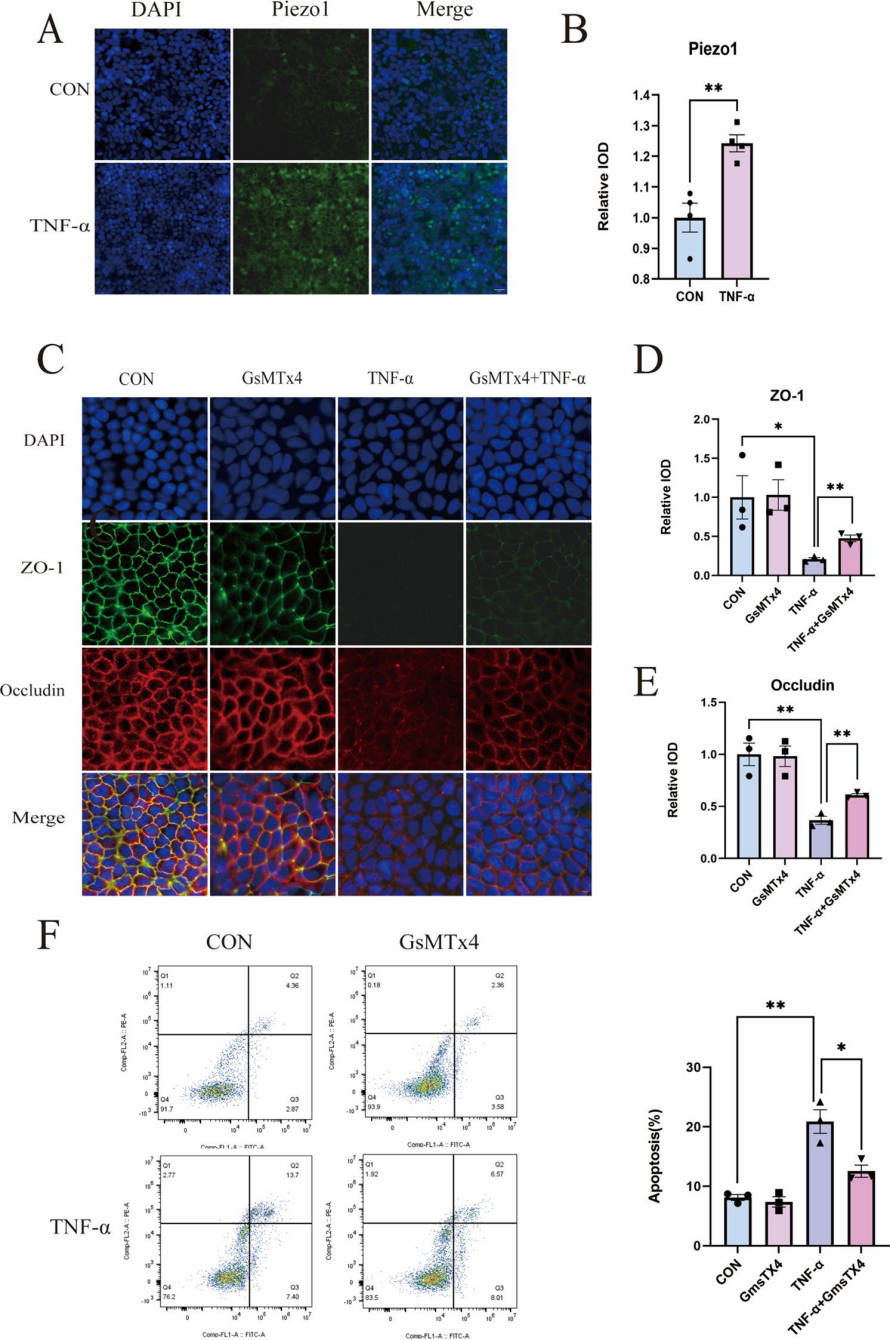
Piezo1 plays a pivotal role in the disruption of the intestinal barrier induced by TNF-α leading to increased degradation of TJ proteins and apoptosis. **A**, **B** Immunofluorescence analysis of Piezo1 Caco-2 cell monolayers, and quantification of immunofluorescence analysis (n = 3 for each group). Scale bar, 50 μm. **C**–**E** Immunofluorescence analysis of ZO1 and Occludin in Caco-2 cell monolayers, and quantitative analysis of immunofluorescence evaluation (n = 3 for each group). Scale bar, 20 μm. **F** Apoptosis were analysis by PE and ANNEXIN V- FITC double staining, and apoptosis rates are displayed as histograms (n = 3 for each group)

Immunostaining of Caco-2 cell monolayers stimulated with TNF-α revealed a substantial Piezo1 expression in the cellular membrane (Fig. 3A, B). Our inquiry into the impact of Piezo1 on barrier function and TJ integrity in these cell monolayers unveiled that Piezo1 triggers the relocation of occludin and ZO-1 away from the epithelial junctions in TNF-α-induced conditions. However, this phenomenon was not observed in cell monolayers treated with GsMTx4, an inhibitor of mechanosensitive channels (Fig. 3C–E). To elucidate Piezo1's involvement in modulating apoptosis during injury to the intestinal epithelial monolayer barrier, we administered TNF-α to induce epithelial apoptosis, both in the presence and absence of functional Piezo1. Utilizing flow cytometry, the apoptotic cells were quantified, and it was found that Piezo1 inhibition led to a decrease in the apoptosis of Caco-2 cells, as evidenced by the diminished presence of PE-positive cells. Additionally, the administration of GsMTx4 effectively restrained the apoptosis of intestinal epithelial cells following TNF-α incubation (Fig. 3F).

### Piezo1 induces mitochondrial impairment in Caco-2 cells in vitro

**Fig. 4:**
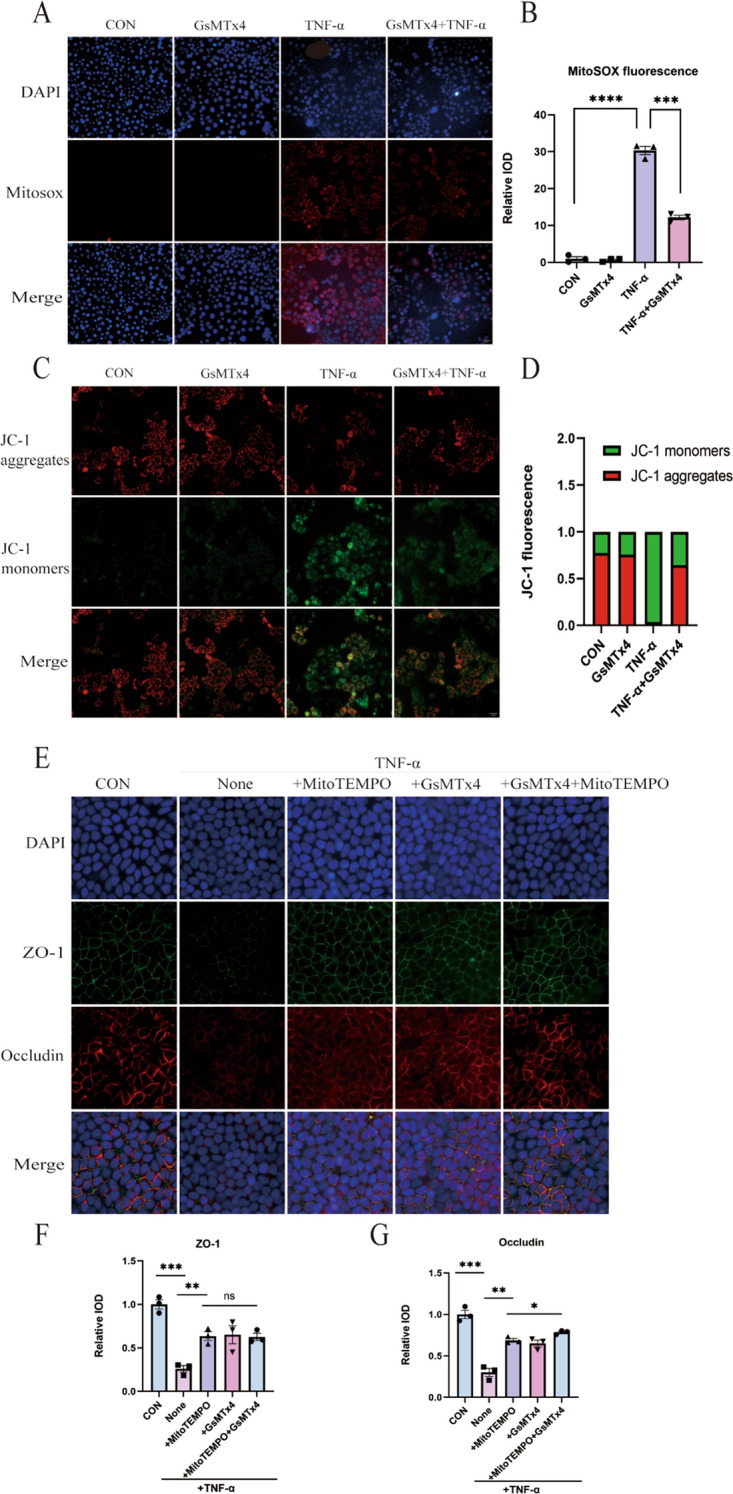
Piezo1 Induces Mitochondrial Impairment in Caco-2 Cells In Vitro. **A**, **B** Measurement of mitochondrial ROS through MitoSOX, along with quantitative analysis of immunofluorescence evaluation (n = 3 for each group). Scale bar, 50 μm. **C**, **D** Assessment of mitochondrial membrane potential using JC-1 probes, with the relative intensity of red fluorescence to green fluorescence was used for quantitative analysis (n = 3 for each group). Scale bar, 50 μm. **E**–**G** Immunofluorescence analysis of ZO1 and Occludin in Caco-2 cell monolayers, accompanied by quantification of immunofluorescence analysis (n = 3 for each group). Scale bar, 20 μm

During inflammation, mitochondrial oxidative energy metabolism plays a pivotal role in maintaining the integrity of the gut epithelial barrier. We investigated the potential effects of Piezo1 on TNF-α-induced mitochondrial dysfunction in Caco-2 cells. Comparative analysis revealed that treatment with GsMTx4, as opposed to TNF-α treatment alone, led to a substantial reduction in mitochondrial dysfunction, manifested through reduced levels of mitochondrial ROS and an enhanced mitochondrial membrane potential. (Fig. 4A–D). To delve deeper into the role of mitochondrial respiration in the TNF-α-mediated regulation of TJs, Caco-2 cells were pre-treated with the mitochondria-targeted antioxidant MitoTEMPO before the administration of TNF-α and GsMTx4. Significantly mirroring the effects of GsMTx4 treatment, pre-treatment with MitoTEMPO distinctly restored the decreased expression of TJs (Fig. 4E–G). Interestingly, the levels of ZO-1 demonstrated similarity between cells treated exclusively with TNF-α and those subjected to TNF-α along with GsMTx4, both in the presence of MitoTEMPO; however, the expression level of occludin was noticeably restored when GsMTx4 and mito-tempo were used in combination.

### Ca^2+^ promoted TNF-α-induced intestinal barrier disruption in Caco-2 cells

**Fig. 5:**
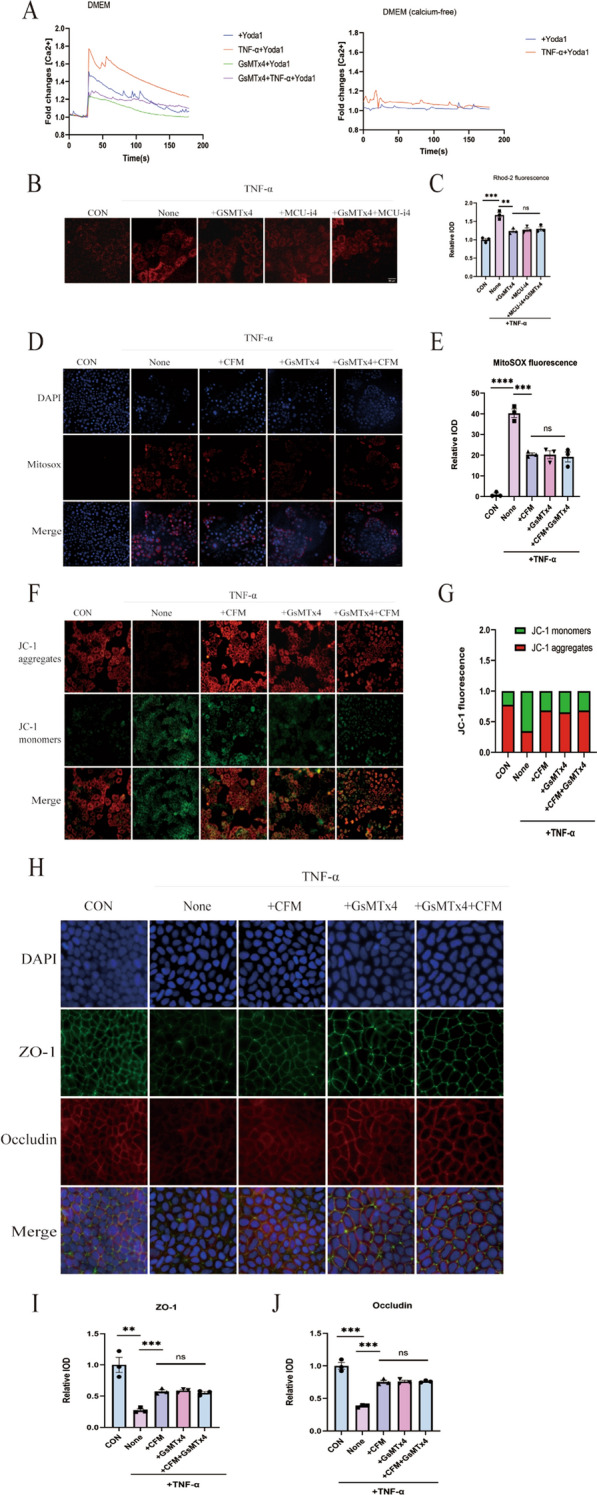
Ca^2+^ Promoted TNF-α-induced intestinal barrier disruption in Caco-2 cells. **A** Calcium imaging of Caco-2 cell (n = 10 for each group). **B**, **C** Mitochondrial ROS were measured by MitoSOX, and quantification of immunofluorescence analysis (n = 3 for each group). Scale bar, 50 μm. **D**, **E** Mitochondrial ROS were measured by MitoSOX, and quantification of immunofluorescence analysis (n = 3 for each group). Scale bar, 50 μm. **F**, **G** Mitochondrial membrane potentia were measured by JC-1 probes, the relative IOD ratio quantitative analysis was performed using the ratio of red to green fluorescence. (n = 3 for each group). Scale bar,50 μm. **H**–**J** Immunofluorescence analysis of ZO1 and Occludin in Caco-2 cell monolayers, and quantification of immunofluorescence analysis (n = 3 for each group). Scale bar, 20 μm

Piezo1 functions as a constituent of the Ca^2+^-permeable nonselective cation channel and is implicated in the regulation of mitochondrial transporters, enzymes, and respiratory complexes. Thus, we hypothesized that intracellular calcium exacerbates intestinal barrier disruption by inducing mitochondrial dysfunction. To investigate this further, we conducted in vitro calcium imaging experiments using Caco-2 cells. Following treatment with Yoda1, a potent Piezo1 agonist, a significant increase in calcium influx was observed, particularly in intestinal epithelial cells, following TNF-α incubation. However, this effect was mitigated by pre-treatment with GsMTx4. Additionally, incubating cells in a calcium-free medium eliminated the Yoda1 and TNF-α-induced elevation in calcium influx (Fig. 5A). The intracellular Ca^2+^ concentration increased and the mitochondrial uptake of the overloaded Ca^2+^ in the matrix through the mitochondrial calcium single transporter (MCU) ultimately induced mitochondrial calcium overload, leading to mitochondrial dysfunction. To determine the change of Ca^2+^ in the mitochondria, we used a Rhod-2 AM probe to detect the concentration of Ca^2+^ in the mitochondria. We found that the increase in intracellular calcium ions caused by the inhibition of TNF-α by Gsmtx4 was accompanied by a corresponding decrease in the concentration of calcium ions in the mitochondria, and a similar effect was produced using the MCU inhibitor mcu-i4 (Fig. 5B, C). Subsequently, we examined the effect of calcium on mitochondrial dysfunction. The use of a calcium-free medium alleviated the mitochondrial dysfunction induced by TNF-α, including the production of mitochondrial ROS and disruption of the mitochondrial membrane potential. This effect was similar to that observed for GsMTx4 (Fig. 5D–G). Furthermore, the redistribution of occludin and ZO-1 from TJs, caused by TNF-α, was also inhibited by the calcium-free medium. Remarkably, the levels of TJ proteins exhibited comparability between cells treated solely with TNF-α and those subjected to TNF-α along with GsMTx4 in the context of a calcium-free medium (Fig. 5H–J). In addition, we used mcu-i4 and observed similar results to GMSTX, such as improvement in mitochondrial dysfunction and reduction in barrier disruption (Additional file 1: Figure S2).

## Discussion

The fundamental basis of the pathophysiology of sepsis lies in the systemic translocation of bacteria, which is intricately intertwined with the complex manifestations of MODS. Maintaining the integrity of the intestinal barrier is of paramount importance in the progression of sepsis. Our investigations indicated that Piezo1 exhibited increased expression in CLP-induced septic mice, showing a correlation with intestinal barrier impairment during sepsis. Piezo1 deficiency in mice ameliorated CLP-induced inflammation and maintained the integrity of the epithelial barrier by attenuating epithelial apoptosis and enhancing the expression of TJs. In our in vitro experiments, we revealed the regulatory function of Piezo1 in controlling calcium influx within Caco-2 cells, ultimately leading to mitochondrial dysfunction characterized by the build-up of mitochondrial ROS and a reduction in membrane potential, which might precipitate the breakdown of TJs.

The intestinal epithelial barrier comprises a monolayer of epithelial cells and TJ proteins, which facilitate intercellular connections, seal the paracellular space, and regulate barrier permeability. Impairment of the intestinal mucosa expedites apoptosis and subsequently exacerbates intestinal permeability [16]. Notably, during ARDS, Piezo1 mediates the apoptosis of type II pneumocytes, as documented by Liang et al. [17]. Liu et al. have demonstrated that Piezo1 plays a pivotal role in inducing AFC apoptosis [18]. To the best of our knowledge, this study marks the first report on Piezo1's role in promoting epithelial apoptosis, thereby regulating the integrity of the intestinal mucosal barrier during CLP. However, further investigation is required to elucidate the precise mechanism by which Piezo1 regulates subsequent processes related to apoptosis.

The TJs of intestinal epithelial cells intricately connect with the intracellular cytoskeleton via various families of intramembrane proteins (e.g., claudin, occludin, tricellulin, and junctional adhesion molecules) and intracellular connexins (e.g., ZO and myosin light chains), thereby upholding the function of the intestinal barrier [19]. Using in vitro experiments, Jiang et al. discovered that Piezo1 adversely regulates epithelial barrier function by influencing the expression of claudin-1 [20]. Importantly, our findings in sepsis revealed that Piezo1-deficient mice maintained the expression of pivotal TJ proteins (ZO-1 and occludin) that are crucial for the integrity of the intestinal mucosal barrier. These in vivo observations were corroborated by our in vitro data, illustrating the attenuation of TJ disruption in cell monolayers following TNF-α incubation upon Piezo1 inhibition.

Previous research has demonstrated the ability of Piezo1 to induce calcium influx [21]. Notably, after pretreatment of Caco-2 cells with TNF-α, calcium influx increased relative to the control group; however, the utilization of Piezo1 inhibitors subsequently reduced TNF-α-mediated calcium influx, indicating the engagement of Piezo1 in calcium influx during inflammation. During sepsis, inflammatory factors activate ion channels in cells, thereby increasing the intracellular Ca^2+^ concentrations [22]. Consequently, the mitochondria accumulate excessive Ca^2+^ in the matrix, leading to mitochondrial calcium overload [23]. This phenomenon triggers the activation of mitochondrial permeability transition pores, ultimately disrupting mitochondrial membrane potential. In addition, augmented mitochondrial Ca^2+^ promotes succinate accumulation, thereby stimulating ROS production [24]. These findings laid the foundation for the present study. Subduing calcium influx leads to diminished mitochondrial oxidant production and restoration of the mitochondrial membrane potential. Curtailing calcium influx concurrently leads to the restoration of cellular TJs.

Nevertheless, it is imperative to acknowledge the limitations of this study. Although animal models have provided invaluable insights, the intricacies of human sepsis have not been comprehensively captured. Translation of our findings from murine experiments to clinical applications requires careful consideration and rigorous validation. Furthermore, a deeper exploration of the precise role of Piezo1 in sepsis-associated inflammation is required.

## Conclusions

In conclusion, our study introduces Piezo1 as a prospective contributor to sepsis-induced intestinal barrier dysfunction, exerting an influence on apoptosis and alteration of tight junctions mediated by calcium influx and mitochondrial dysfunction. The outcomes of our study potentially open avenues for novel therapeutic strategies in sepsis management, while expanding the comprehension of Piezo1's broader implications in cellular physiology and pathology.

### Supplementary Information

**Additional file 1: Figure S1.** The changes of microbiota in mice with or without CLP. **Figure S2.** Effect of MCU-i4 on Mitochondrial Dysfunction and Tight Junctions.

## Data Availability

All data generated or analysed during this study are included in this published article.

## Abbreviations

WT: Wild-type
CLP: Cecal ligation and puncture
TJs: Tight junctions
RT-PCR: Real-time PCR
IL: Interleukin
CFM: Calcium-free medium
LPS: Lipopolysaccharide
FBS: Fetal Bovine Serum
PBS: Phosphate Buffered Saline
