# Supplementary material for: Intestinal Piezo1 aggravates intestinal barrier dysfunction during sepsis by mediating Ca2+ influx
Source: J Transl Med. 2024 Apr 4;22:332. doi: 10.1186/s12967-024-05076-z (PMC10996241; doi:10.1186/s12967-024-05076-z)
Supplement: Supplementary file 1 — Additional file 1: Figure S1. The changes of microbiota in mice with or without CLP. Figure S2. Effect of MCU-i4 on Mitochondrial Dysfunction and Tight Junctions. [file 12967_2024_5076_MOESM1_ESM.pdf]

Intestinal Piezo1 aggravates intestinal barrier dysfunction during sepsis by mediating Ca^{2+} influx

Authors: Zimeng Yan[1f], Lei Niu[2f], Shangyuan Wang[1f], Chengjin Gao[1*], Shuming Pan[1*].

1. Department of Emergency, Xinhua Hospital Affiliated to Shanghai Jiao Tong University School of Medicine, Yangpu District, Shanghai, China

2. Department of Emergency, Shanghai Jiahui International Hospital, Shanghai, China. No. 689, Guiping Rd. Shanghai, China.

Correspondence:

Chengjin Gao

gaochengjin@xinhuamed.com

Shuming Pan

Panpanq123q@163.com

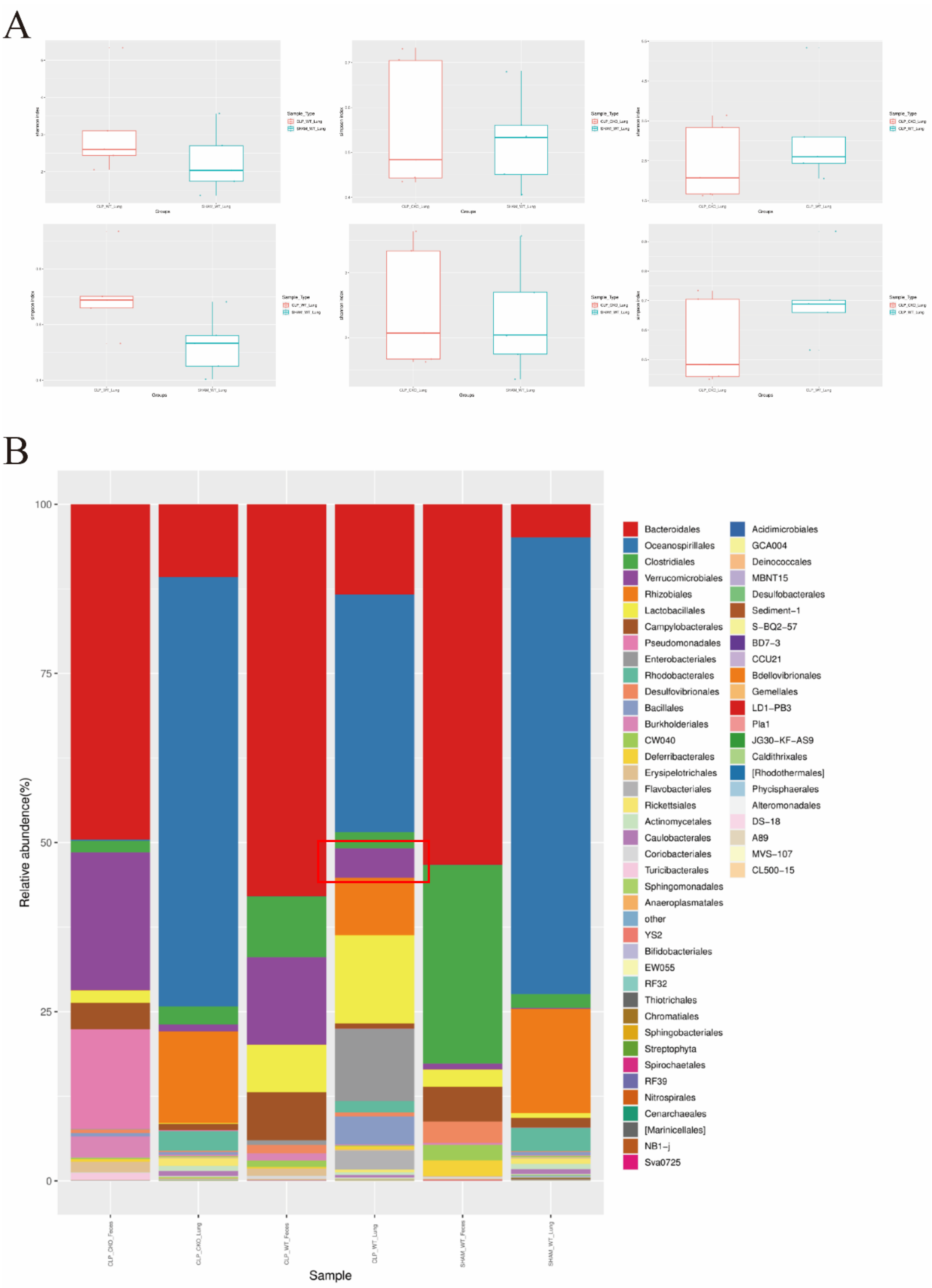

Fig.S1 The changes of microbiota in mice with or without CLP. (A). The α diversity of the pulmonary microbiota in mouse lung tissue (n = 5 for each group). (B). Taxonomic compositions of samples at Order level inmice feces and lung tissue (n = 5 for each group).

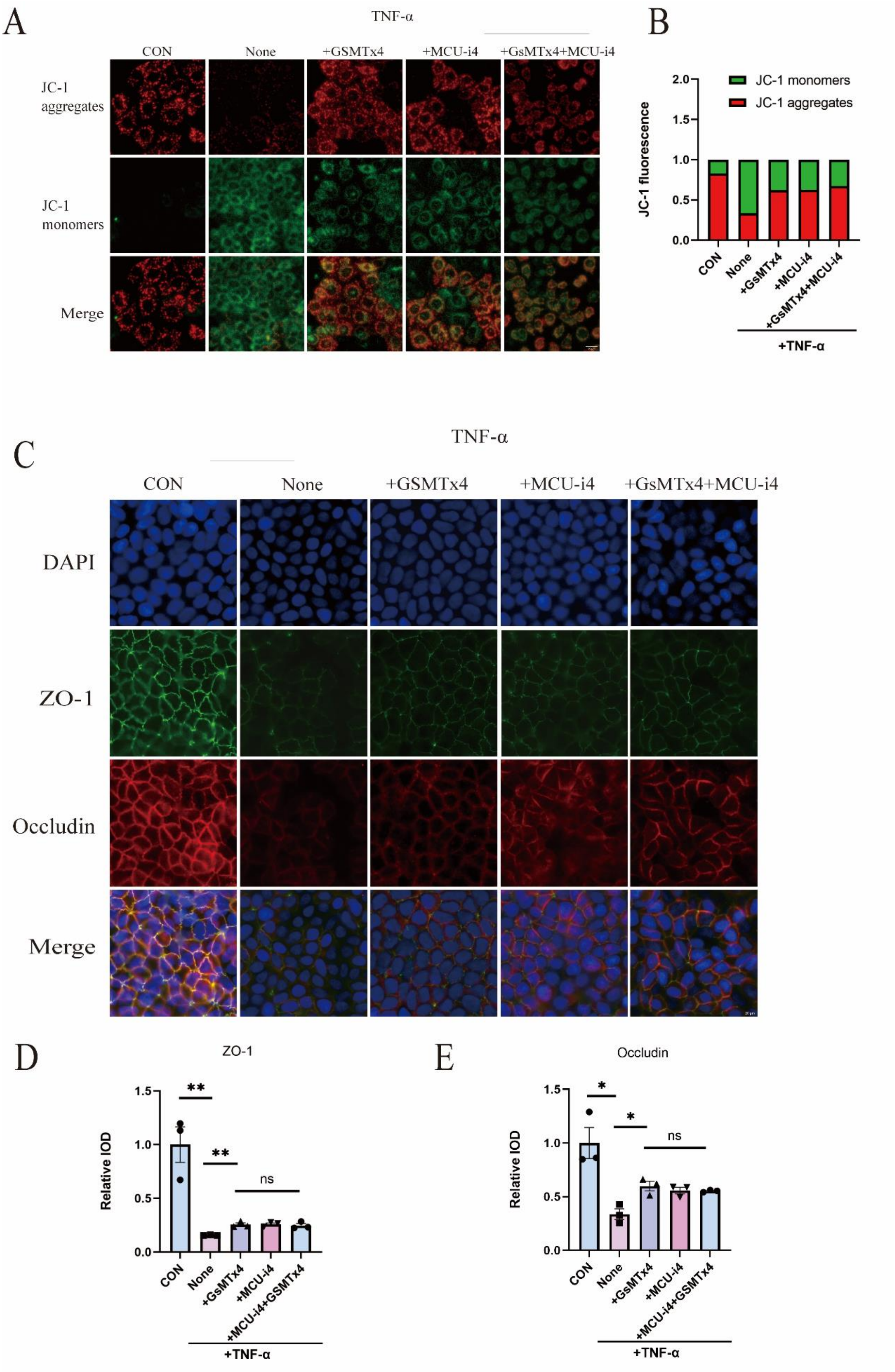

Fig.2s Effect of MCU-i4 on Mitochondrial Dysfunction and Tight Junctions. (A-B). Mitochondrial membrane potentia were measured by JC-1 probes, the relative IOD ratio quantitative analysis

was performed using the ratio of red to green fluorescence. (n = 3 for each group). Scale bar,50 μm. (C-E). Immunofluorescence analysis of ZO1 and Occludin in Caco-2 cell monolayers, and quantification of immunofluorescence analysis (n = 3 for each group). Scale bar, 20μm.
